# Variation of Risk Factors for Cause-Specific Reintubation: A Preliminary Study

**DOI:** 10.1155/2018/3654251

**Published:** 2018-10-28

**Authors:** Emi Fujii, Kazunori Fujino, Sachiko Tanaka-Mizuno, Yutaka Eguchi

**Affiliations:** ^1^Department of Critical and Intensive Medicine, Shiga University of Medical Science, Seta-tsukinowa-cho, Otsu-shi, Shiga 527-8505, Japan; ^2^Department of Medical Statistics, Shiga University of Medical Science, Seta-tsukinowa-cho, Otsu-shi, Shiga 527-8505, Japan

## Abstract

Unexpected reintubation may occur, even if the risk factors are considered and a spontaneous breathing trial is successful. Reintubation is thought to be caused by various factors. Several studies have investigated the risk factors of reintubation, but most did not classify reintubation by cause. We retrospectively classified patients undergoing reintubation at intensive care unit by cause (respiratory insufficiency vs. nonrespiratory insufficiency) to examine the cause-specific risk factors of reintubation. A total of 262 patients were included; reintubation within 48 hours after extubation was performed in 12 patients (reintubation rate, 4.5%). After classification by cause of reintubation, the pressure of arterial oxygen to fractional inspired oxygen concentration (*P*/*F*) ratio exhibited a significant association with reintubation only in the respiratory insufficiency group (odds ratio (OR) 0.989, 95% confidence interval (CI) 0.980 to 0.999, *p*=0.036, and OR 0.989, 95% CI 0.979 to 0.999, *p*=0.026, in the univariate and multivariate analyses, respectively). In the propensity score analysis, a *P*/*F* ratio ≤ 200 may be a risk factor for reintubation in the respiratory insufficiency group (OR 7.811, 95% CI 1.345 to 45.367, *p*=0.022). In the nonrespiratory insufficiency group, intubation duration was significantly related to reintubation (OR 1.165, 95% CI 1.012 to 1.342, *p*=0.033, and OR 1.163, 95% CI 1.004 to 1.348, *p*=0.044, in the univariate and multivariate analyses, respectively). In conclusion, a low *P*/*F* ratio at extubation may be a risk factor for reintubation due to respiratory insufficiency. In the nonrespiratory insufficiency group, intubation duration may be significantly related to reintubation. The risk factors for reintubation may differ by the cause of reintubation. Further large-scale randomized controlled trials are required.

## 1. Introduction

In the intensive care unit (ICU), approximately 30% of all patients require mechanical ventilation to assist respiration [1]. Long-term ventilation can lead to complications such as ventilator-associated pneumonia and ventilator-associated lung injury, which greatly affect the length of stay and mortality in the ICU. Moreover, ventilator days are an independent risk factor of mortality in the ICU [2, 3]. However, extubation failure is associated with poor outcomes, including high mortality [4–8].

The use of a ventilator weaning protocol for the performance of a spontaneous breathing trial (SBT) [9] has been widely recommended for extubation from ventilation. Although this strategy is generally successful, reintubation is required in ≥15% of patients [6, 10, 11]. To reduce the rate of reintubation, it is necessary to accurately evaluate the risk factors.

Reintubation is thought to be caused by various factors. To reduce reintubation rates, several studies have been conducted, and several risk factors (age, Acute Physiology and Chronic Health Evaluation II (APACHE II) scores [12], intubation duration [12], rapid shallow breathing index (RSBI) [13], and positive fluid balance on the day before extubation [10]) have been reported. However, to the best of our knowledge, no study has investigated the risk factors of reintubation after classifying the reintubation cases by cause. The predominant causes of reintubation are respiratory insufficiency, upper airway factors such as laryngeal complications, lowered level of consciousness, and haemodynamic instability. To assess upper airway factors, the cuff leak test is commonly performed; however, this procedure has low sensitivity and cannot predict reintubation [14].

In this study, we classified reintubation subjects into those with respiratory insufficiency and those with nonrespiratory insufficiency and retrospectively investigated the risk factor of reintubation by cause.

## 2. Materials and Methods

### 2.1. Ethical Approval

This study was approved by the Ethics Committee of the Shiga University, Medical Science Hospital, Shiga, Japan. Informed consent of the patients was obtained (approval number: 28-17). The work complied with the Helsinki Declaration of 1975 and its subsequent revisions.

### 2.2. Setting

We retrospectively examined adult patients admitted to the ICU of the Shiga University, Medical Science Hospital, Shiga, Japan, between April 2013 and July 2015. In this ICU, both medical and surgical patients are admitted. Cases of cardiovascular surgery were excluded. We investigated 262 patients who were intubated, invasively mechanically ventilated for more than 24 hours, and extubated.

### 2.3. Extubation Criteria

In our hospital's ICU, the extubation criteria include a successful SBT and a confirmed negative cuff leak test. The method and period of the SBT are left to the discretion of the attending physician. The respiratory settings of the SBT include a continuous positive airway pressure with a positive end-expiratory pressure of 4 cm H_2_O and a pressure support of 4 cm H_2_O; sometimes, T-pieces are used.

### 2.4. Reintubation Criteria

When the attending physician decided that reintubation is necessary, reintubation was carried out. Deterioration of mental state such as agitation, haemodynamic instability (tachycardia, arrhythmia, and elevation of blood pressure), increased respiratory rate, use of respiratory support muscle, decrease in partial pressure of arterial oxygen (PaO_2_), and increase in partial pressure of arterial carbon dioxide (PaCO_2_) were considered to be indicators for the requirement of reintubation [15].

### 2.5. Measures and Classification

We examined the subjects' age, sex, Acute Physiology and Chronic Health Evaluation II (APACHE II) and Sequential Organ Failure Assessment (SOFA) scores at admission, the intubation duration, pressure of arterial oxygen to fractional inspired oxygen concentration ratio (PaO_2_/FiO_2_ ratio or *P*/*F* ratio), PaCO_2_, rapid shallow breathing index (RSBI) at extubation, and positive fluid balance on the day before extubation. Moreover, we investigated the use of noninvasive ventilation (NIV) after extubation and the prognoses (28- and 90-day mortality) associated with reintubation.

Subjects in whom reintubation was required within 48 hours after extubation were defined as reintubation cases. Subsequently, the reintubation cases were divided into two groups, according to the cause of reintubation. Respiratory muscle fatigue, excessive airway secretion, a weak cough, hypoxaemia, and hypercapnia were defined as respiratory insufficiency, whereas upper airway factors (laryngeal oedema, mucosal ulcers, granulation, and vocal cord paralysis), haemodynamic instability, and lowered level of consciousness were defined as nonrespiratory insufficiency (Table 1).

### 2.6. Statistical Analyses

The baseline characteristics of the subjects are shown as means and standard deviations for continuous variables and numbers and proportions for categorical variables. We compared the baseline characteristics between the successful extubation and reintubation groups using the chi-square and Mann–Whitney *U*-tests. The reintubation group was further classified into the respiratory insufficiency and nonrespiratory insufficiency subgroups. We conducted univariate and age-adjusted multivariate logistic regression to identify the risk factors for reintubation. Odds ratios (ORs) and 95% confidence intervals (CIs) were calculated; *p* < 0.05 was considered significant.

After dividing the subjects into the two groups according to the cause of reintubation, we used the propensity score method to adjust for possible confounders. First, to investigate the risk factors of reintubation due to respiratory insufficiency, we added the candidate confounders age, APACHE II score, intubation duration, and RSBI to a logistic regression model with reintubation as the dependent variable. These confounders were previously reported to be risk factors for reintubation [8, 10, 12, 16–18]. In the final model, we conducted logistic regression including a *P*/*F* ratio ≤ 200, the propensity scores as covariates, and reintubation as the dependent variable. In past reports, a *P*/*F* ratio > 150 was recommended as the SBT initiation criterion [19–22]; thus, we used a *P*/*F* ratio ≤ 200 as a covariate in this study. Since the number of cases in the nonrespiratory insufficiency groups was small, we did use propensity score analysis for this group. All statistical analyses were performed with SPSS Statistics Version 22 (IBM Japan, Tokyo, Japan).

## 3. Results

### 3.1. Subject Characteristics and Causes of Reintubation

**Figure 1:**
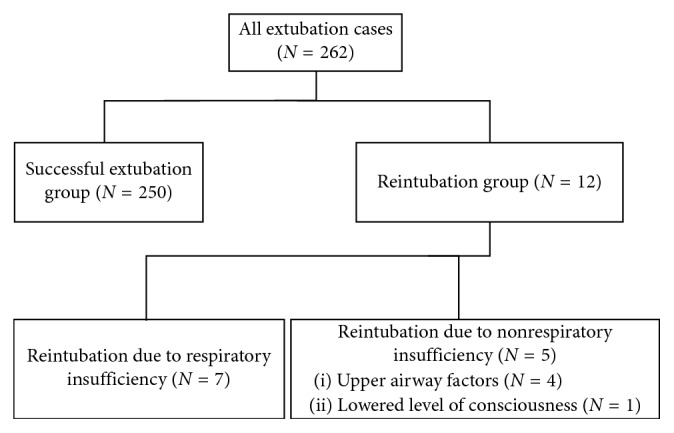
Flowchart.

During the study period, 262 patients were admitted to the ICU and extubated. Of these, 250 were successfully extubated, and 12 were reintubated within 48 hours after extubation (reintubation rate, 4.5%; Figure 1).

There were no significant differences in age, sex, APACHE II scores, and SOFA scores between the successful extubation and reintubation groups. The intubation duration was significantly longer (4.5 days vs. 1.0 day, respectively; *p*=0.006), and the RSBI was significantly higher (63.0 breaths/min/L vs. 43.0 breaths/min/L, respectively; *p*=0.035) in the reintubation group than in the extubation group (Table 2). There were no significant differences in PaCO_2_ at extubation between the groups. Noninvasive ventilation was performed in 28 out of all patients and 4 patients from all reintubation groups (2 from the respiratory insufficiency group and 2 from the nonrespiratory insufficiency group). In the reintubation group, we did not observe any complications such as ventilator-associated pneumonia or ventilator-associated lung injury. Moreover, no deterioration of the prognoses (28- and 90-day mortality) was observed.

### 3.2. Risk Factors of Reintubation by Cause

Among 12 subjects requiring reintubation, the cause was respiratory insufficiency and nonrespiratory insufficiency in 7 and 5 cases (4 due to upper airway factors and 1 due to lowered level of consciousness), respectively (Figure 1).

In both univariate and multivariate analyses, after adjustment for age, intubation duration and RSBI were significantly associated with reintubation in the total reintubation group (univariate analyses: intubation duration: OR 1.128, 95% CI 1.018 to 1.249, and RSBI: OR 1.034, 95% CI 1.006 to 1.063; multivariate analyses: intubation duration: OR 1.123, 95% CI 1.013 to 1.246, and RSBI: OR 1.033, 95% CI 1.005 to 1.061; Table 3). Moreover, the *P*/*F* ratio exhibited a significant association with reintubation, but only in the respiratory insufficiency group (*p*=0.036 and *p*=0.026 in the univariate and multivariate analyses, respectively). Intubation duration was significantly related to reintubation in the nonrespiratory insufficiency group only (*p*=0.033 and *p*=0.044 in the univariate and multivariate analyses, respectively; Table 3).

### 3.3. Propensity Score Analysis

In the reintubation group, a *P*/*F* ratio ≤ 200 at extubation was not associated with reintubation in the propensity score analysis after adjustments for age, APACHE score, intubation duration, and RSBI. However, in the respiratory insufficiency group, a *P*/*F* ratio ≤ 200 showed a significant association with reintubation (*P*/*F* ratio ≤ 200 at extubation: OR 7.811, 95% CI 1.345 to 45.367; Table 4). In the nonrespiratory insufficiency group, no patient had a *P*/*F* ratio ≤ 200.

## 4. Discussion

We retrospectively investigated patients who required reintubation despite a successful SBT and cuff leak test at extubation. To clarify the cause-specific risk factors of reintubation, we divided the reintubation subjects into the respiratory insufficiency and nonrespiratory insufficiency groups. We used propensity score analysis to adjust for confounding factors. The odds of reintubation significantly increased with a *P*/*F* ratio ≤ 200 at extubation in the respiratory insufficiency group. In the nonrespiratory insufficiency group, intubation duration may be related to reintubation. Larger-scale studies are needed, but when classified by cause, reintubation seems to have a different risk factor.

The reason a low *P*/*F* ratio at extubation has not been considered as a predictor of reintubation is likely that reintubation cases have previously not been classified by the cause of reintubation. The *P*/*F* ratio at extubation has been disregarded as a risk factor for reintubation [10, 12, 16]. Thille et al. examined 168 patients who were extubated as scheduled and reported that although the *P*/*F* ratio was investigated, there were no significant differences between successful and failed extubation [17]. In our study, the *P*/*F* ratio at extubation was not associated with reintubation in the total reintubation group in the multivariate analysis. This is consistent with the results of previous studies.

However, a low *P*/*F* ratio significantly increased the risk of reintubation when the cause was respiratory insufficiency. Because a deterioration in the *P*/*F* ratio at extubation was not often observed in the nonrespiratory insufficiency group, the *P*/*F* ratio at extubation may not be associated with reintubation when a simultaneous analysis was performed.

Only in the nonrespiratory insufficiency group, the intubation duration significantly increased the risk of reintubation. Penuelas et al. reported that a long duration of intubation at extubation increased the risk of reintubation [12]. In our study, the intubation duration in the reintubation group was longer than that in successful extubation groups, but we observed no significant differences between the successful extubation groups and the respiratory insufficiency group, and the intubation duration was significantly longer in the nonrespiratory insufficiency group than in the successful extubation group. The intubation duration has also been reported as a risk factor of laryngeal injuries after intubation [23], which may explain why the intubation duration was a risk factor for reintubation in the nonrespiratory insufficiency group.

RSBI may not be a risk factor of reintubation when stratified by cause. It was reported that RSBI has high reliability as a predictor of weaning [24], but it is affected by the ventilator support setting [25], so RSBI alone may be insufficient as a predictor. In our survey, a significant difference in the RSBI was found between the reintubation and successful extubation groups. However, when we further classified the reintubation groups into two subgroups by cause, we observed no significant differences in the RSBI between the groups. This is possibly because classification into two groups decreases the number of subjects in each group; this may attenuate the effects of differences observed. It is necessary to investigate by increasing the number of cases.

In this study, it was suggested that cause-specific risk factors might exist upon classifying the cause of reintubation. Although it may be done empirically, there is a possibility that, by classifying the cause, effective treatment for preventing reintubation can be provided. For example, in the nonrespiratory insufficiency group, for the prevention of postextubation laryngeal oedema if corticosteroids administration is provided not to all intubation patients, but only to the patients with long intubation duration, it may be more effective and side effects may be reduced. Patients which need reintubation due to upper airway factors in nonrespiratory insufficiency may be difficult to intubate and should be prepared for difficult airway. Although it was reported that NIV was not effective for postextubation respiratory failure [26], there are reports that early NIV was effective for the prevention of respiratory failure after extubation [27]. Even in patients with a low *P*/*F* ratio at extubation, reintubation may be avoided by providing NIV after extubation. By increasing the number of cases and classifying them according to the cause, the risk factors of reintubation will become clearer. It will also contribute to the development of a treatment regimen corresponding to each disease condition.

Our study has several limitations. First, the number of reintubations was low initially and further decreased due to the cause-specific classification. Second, since reintubation was caused by a variety of factors, an accurate classification was difficult. There was no case in this study, but there may be patients with both respiratory insufficiency and upper airway factors.

Third, while we examined and adjusted for age, APACHE II score, intubation duration, and RSBI, airway secretions and a weak cough reflex, which are also risk factors of reintubation, were difficult to investigate, and an objective evaluation of these factors was difficult. Fourth, this study had a single-centre retrospective design. Finally, all subjects were of Asian descent; thus, possible differences associated with ethnicity could not be considered.

## 5. Conclusions

When we investigated the risk factors of reintubation by cause, we found that a low *P*/*F* ratio at extubation in patients with respiratory insufficiency was a predictor of reintubation. Moreover, the intubation duration might be a risk factor in patients with nonrespiratory insufficiency. This was a small-scale retrospective study; although large studies may show different results, our results indicate that the risk factors vary between reintubation due to respiratory failure and reintubation due to nonrespiratory failure. By increasing the sample size and examining the cause-specific risks of reintubation, the risk of reintubation can be predicted with a higher accuracy. This can aid in the development of treatment strategies corresponding to each pathophysiology.

## Figures and Tables

**Table 1:** Classification of the causes of reintubation.

Respiratory insufficiency group		Respiratory muscle fatigue
		Excessive airway secretion
		Weak cough
		Hypoxaemia
		Hypercapnia

Nonrespiratory insufficiency group	Upper airway factors	Laryngeal oedema
		Mucosal ulcer, granulation
		Vocal cord paralysis
	Lowered level of consciousness	
	Haemodynamic instability	

**Table 2:** Characteristics of the subjects.

	All	Successful extubation group	Reintubation group					
			Total	*p* value	Respiratory insufficiency group	*p* value	Nonrespiratory insufficiency group	*p* value
Patients, *n* (%)	262	250 (95.4)	12 (4.5)		7 (2.6)		5 (1.9)	
Age	64.0 ± 16.2	65.0 ± 16.1	60.0 ± 16.8	0.151	62.0 ± 18.2	0.808	43.0 ± 13.3	0.053
Sex (male : female)	149 : 113	144 : 106	5 : 7	0.276	4 : 3	0.981	1 : 4	0.093
APACHE score	12.5 ± 7.7	12.0 ± 7.5	17.5 ± 9.1	0.257	17.0 ± 8.2	0.626	18.0 ± 11.1	0.230
SOFA score	6.0 ± 2.9	6.0 ± 2.9	5.0 ± 2.9	0.979	7.0 ± 2.7	0.960	5.0 ± 3.5	0.988
Intubation duration (days)	1.0 ± 4.0	1.0 ± 3.9	4.5 ± 5.0	0.006	4.0 ± 6.0	0.253	9.0 ± 2.9	0.003
*P*/*F* ratio	350.0 ± 89.2	360.0 ± 88.3	341.0 ± 106.8	0.327	252.0 ± 106.6	0.056	350.0 ± 81.2	0.440
RSBI	43.0 ± 17.0	43.0 ± 16.6	63.0 ± 20.6	0.03	58.0 ± 19.0	0.106	68.0 ± 24.7	0.175
The positive fluid balance (ml)	85.0 ± 1082.5	103.1 ± 1088.6	−291.5 ± 902.7	0.141	67.0 ± 580.6	0.734	−793.4 ± 1093.3	0.056
PaCO_2_ (mmHg)	39.7 ± 5.0	39.5 ± 4.9	42.8 ± 6.4	0.119	42.6 ± 8.2	0.478	42.9 ± 3.7	0.108
28-day mortality, *n* (%)	2 (0.7)	2 (0.7)	0 (0)	0.756	0 (0)	0.812	0 (0)	0.842
90-day mortality, *n* (%)	14 (5.3)	14 (5.3)	0 (0)	0.399	0 (0)	0.520	0 (0)	0.592

Values are expressed as means ± standard deviations or numbers (percentages). The *p* value of each group is described in comparison to the successful extubation group. APACHE: acute physiology and chronic health evaluation; SOFA: sequential organ failure assessment; *P*/*F* ratio: pressure of arterial oxygen to fractional inspired oxygen concentration ratio; RSBI: rapid shallow breathing index; PaCO_2_: partial pressure of arterial carbon dioxide.

**Table 3:** Univariate and age-adjusted multivariate logistic regression analysis by cause of reintubation.

	Univariate logistic regression			Multivariate regression (age-adjusted)		
	OR	95% CI	*p* value	OR	95% CI	*p* value
*Reintubation group*						
Age	0.977	0.944 to 1.011	0.185			
APACHE score	1.055	0.986 to 1.129	0.120	1.066	0.996 to 1.141	0.066
Intubation duration	1.128	1.018 to 1.249	0.022	1.123	1.013 to 1.246	0.028
*P*/*F* ratio	0.996	0.989 to 1.003	0.228	0.994	0.987 to 1.001	0.105
RSBI	1.034	1.006 to 1.063	0.017	1.033	1.005 to 1.061	0.023

*Respiratory insufficiency group*						
Age	0.995	0.950 to 1.041	0.820			
APACHE score	1.062	0.967 to 1.165	0.208	1.068	0.972 to 1.173	0.173
Intubation duration	1.088	0.947 to 1.250	0.232	1.088	0.946 to 1.250	0.236
*P*/*F* ratio	0.989	0.980 to 0.999	0.036	0.989	0.979 to 0.999	0.026
RSBI	1.030	0.994 to 1.067	0.106	1.030	0.994 to 1.067	0.108

*Nonrespiratory insufficiency group*						
Age	0.959	0.913 to 1.007	0.092			
APACHE score	1.083	0.982 to 1.195	0.111	1.108	0.998 to 1.229	0.054
Intubation duration	1.165	1.012 to 1.342	0.033	1.163	1.004 to 1.348	0.044
*P*/*F* ratio	1.004	0.994 to 1.013	0.449	1.002	0.992 to 1.012	0.734
RSBI	1.038	0.998 to 1.080	0.065	1.033	0.994 to 1.074	0.097

APACHE, acute physiology and chronic health evaluation; CI, confidence interval; OR, odds ratio; *P*/*F* ratio, pressure of arterial oxygen to fractional inspired oxygen concentration ratio; RSBI, rapid shallow breathing index.

**Table 4:** Propensity score analysis in the respiratory insufficiency group.

*P*/*F* ≤ 200	Logistic regression using propensity scores (adjusted for age, APACHE score, intubation duration, and RSBI)		
	OR	95% CI	*p* value
Reintubation group	4.004	0.776 to 20.664	0.098
Respiratory insufficiency group	7.811	1.345 to 45.367	0.022

APACHE, acute physiology and chronic health evaluation; CI, confidence interval; OR, odds ratio; *P*/*F* ratio, pressure of arterial oxygen to fractional inspired oxygen concentration ratio; RSBI, rapid shallow breathing index.

## Data Availability

The data used to support the findings of this study are available from the corresponding author upon request.

## References

[1] Esteban A., Anzueto A., Frutos F. (2002). Characteristics and outcomes in adult patients receiving mechanical ventilation: a 28-day international study. *JAMA*.

[2] Mancebo J. (1996). Weaning from mechanical ventilation. *European Respiratory Journal*.

[3] Dries D. J. (1997). Weaning from mechanical ventilation. *Journal of Trauma: Injury, Infection, and Critical Care*.

[4] Epstein S. K., Ciubotaru R. L., Wong J. B. (1997). Effect of failed extubation on the outcome of mechanical ventilation. *Chest*.

[5] Thille A. W., Richard J. C., Brochard L. (2013). The decision to extubate in the intensive care unit. *American Journal of Respiratory Critical Care Medicine*.

[6] Esteban A., Alia I., Gordo F. (1997). Extubation outcome after spontaneous breathing trials with T-tube or pressure support ventilation. *American Journal of Respiratory Critical Care Medicine*.

[7] Epstein S. K., Ciubotaru R. L. (1998). Independent effects of etiology of failure and time to reintubation on outcome for patients failing extubation. *American Journal of Respiratory Critical Care Medicine*.

[8] Upadya A., Tilluckdharry L., Muralidharan V., Amoateng-Adjepong Y., Manthous C. A. (2005). Fluid balance and weaning outcomes. *Intensive Care Medicine*.

[9] Blackwood B., Alderdice F., Burns K., Cardwell C., Lavery G., O’Halloran P. (2011). Use of weaning protocols for reducing duration of mechanical ventilation in critically ill adult patients: cochrane systematic review and meta-analysis. *BMJ*.

[10] Frutos-Vivar F., Ferguson N. D., Esteban A. (2006). Risk factors for extubation failure in patients following a successful spontaneous breathing trial. *Chest*.

[11] Thille A. W., Boissier F., Ghezala H., Razazi K., Mekontso-Dessap A., Brun-Buisson C. (2015). Risk factors for and prediction by caregivers of extubation failure in ICU patients: a prospective study. *Critical Care Medicine*.

[12] Penuelas O., Frutos-Vivar F., Fernandez C. (2011). Characteristics and outcomes of ventilated patients according to time to liberation from mechanical ventilation. *American Journal of Respiratory Critical Care Medicine*.

[13] Yang K. L., Tobin M. J. (1991). A prospective study of indexes predicting the outcome of trials of weaning from mechanical ventilation. *New England Journal of Medicine*.

[14] Ochoa M. E., Marin Mdel C., Frutos-Vivar F. (2009). Cuff-leak test for the diagnosis of upper airway obstruction in adults: a systematic review and meta-analysis. *Intensive Care Medicine*.

[15] Boles J. M., Bion J., Connors A. (2007). Weaning from mechanical ventilation. *European Respiratory Journal*.

[16] Mokhlesi B., Tulaimat A., Gluckman T. J., Wang Y., Evans A. T., Corbridge T. C. (2007). Predicting extubation failure after successful completion of a spontaneous breathing trial. *Respiratory Care*.

[17] Thille A. W., Harrois A., Schortgen F., Brun-Buisson C., Brochard L. (2011). Outcomes of extubation failure in medical intensive care unit patients. *Critical Care Medicine*.

[18] Bien M. Y., Hseu S. S., Yien H. W. (2004). Breathing pattern variability: a weaning predictor in postoperative patients recovering from systemic inflammatory response syndrome. *Intensive Care Medicine*.

[19] Ely E. W., Baker A. M., Dunagan D. P. (1996). Effect on the duration of mechanical ventilation of identifying patients capable of breathing spontaneously. *New England Journal of Medicine*.

[20] Kollef M. H., Shapiro S. D., Silver P. (1997). A randomized, controlled trial of protocol-directed versus physician-directed weaning from mechanical ventilation. *Critical Care Medicine*.

[21] Marelich G. P., Murin S., Battistella F., Inciardi J., Vierra T., Roby M. (2000). Protocol weaning of mechanical ventilation in medical and surgical patients by respiratory care practitioners and nurses: effect on weaning time and incidence of ventilator-associated pneumonia. *Chest*.

[22] Rose L., Presneill J. J., Johnston L., Cade J. F. (2008). A randomised, controlled trial of conventional versus automated weaning from mechanical ventilation using SmartCare/PS. *Intensive Care Medicine*.

[23] Tadie J. M., Behm F., Lecuyer L. (2010). Post-intubation laryngeal injuries and extubation failure: a fiberoptic endoscopic study. *Intensive Care Medicine*.

[24] Meade M., Guyatt G., Cook D. (2001). Predicting success in weaning from mechanical ventilation. *Chest*.

[25] El-Khatib M. F., Zeineldine S. M., Jamaleddine G. W. (2008). Effect of pressure support ventilation and positive end expiratory pressure on the rapid shallow breathing index in intensive care unit patients. *Intensive Care Medicine*.

[26] Agarwal R., Aggarwal A. N., Gupta D., Jindal S. K. (2007). Role of noninvasive positive-pressure ventilation in postextubation respiratory failure: a meta-analysis. *Respiratory Care*.

[27] Ferrer M., Valencia M., Nicolas J. M., Bernadich O., Badia J. R., Torres A. (2006). Early noninvasive ventilation averts extubation failure in patients at risk: a randomized trial. *American Journal of Respiratory and Critical Care Medicine*.

